# Supplementary material for: Prevalence and risk factors of early postoperative seizures in patients with glioma: A protocol for meta-analysis and systematic review
Source: PLoS One. 2024 Apr 4;19(4):e0301443. doi: 10.1371/journal.pone.0301443 (PMC10994364; doi:10.1371/journal.pone.0301443)
Supplement: S2 File — (DOCX) [file pone.0301443.s003.docx]

PubMed

| Search number | Query | Results |
| --- | --- | --- |
| 1 | "Risk Factors"[Mesh] | 951,909 |
| 2 | ((((((((((((((((((Risk Factors[Title/Abstract]) OR (Factor, Risk[Title/Abstract])) OR (Risk Factor[Title/Abstract])) OR (Social Risk Factors[Title/Abstract])) OR (Factor, Social Risk[Title/Abstract])) OR (Factors, Social Risk[Title/Abstract])) OR (Risk Factor, Social[Title/Abstract])) OR (Risk Factors, Social[Title/Abstract])) OR (Social Risk Factor[Title/Abstract])) OR (Health Correlates[Title/Abstract])) OR (Correlates, Health[Title/Abstract])) OR (Population at Risk[Title/Abstract])) OR (Populations at Risk[Title/Abstract])) OR (Risk Scores[Title/Abstract])) OR (Risk Score[Title/Abstract])) OR (Score, Risk[Title/Abstract])) OR (Risk Factor Scores[Title/Abstract])) OR (Risk Factor Score[Title/Abstract])) OR (Score, Risk Factor[Title/Abstract]) | 786,740 |
| 3 | #1 OR #2 | 1,378,675 |
| 4 | "Glioma"[Mesh] | 97,881 |
| 5 | (((((((((((((Glioma[Title/Abstract]) OR (Gliomas[Title/Abstract])) OR (Glial Cell Tumors[Title/Abstract])) OR (Glial Cell Tumor[Title/Abstract])) OR (Tumor, Glial Cell[Title/Abstract])) OR (Tumors, Glial Cell[Title/Abstract])) OR (Mixed Glioma[Title/Abstract])) OR (Glioma, Mixed[Title/Abstract])) OR (Gliomas, Mixed[Title/Abstract])) OR (Mixed Gliomas[Title/Abstract])) OR (Malignant Glioma[Title/Abstract])) OR (Glioma, Malignant[Title/Abstract])) OR (Gliomas, Malignant[Title/Abstract])) OR (Malignant Gliomas[Title/Abstract]) | 70,251 |
| 6 | #4 OR #5 | 119,009 |
| 7 | "Epilepsy"[Mesh] | 124,784 |
| 8 | (((((((((((Epilepsy[Title/Abstract]) OR (Epilepsies[Title/Abstract])) OR (Seizure Disorder[Title/Abstract])) OR (Seizure Disorders[Title/Abstract])) OR (Awakening Epilepsy[Title/Abstract])) OR (Epilepsy, Awakening[Title/Abstract])) OR (Epilepsy, Cryptogenic[Title/Abstract])) OR (Cryptogenic Epilepsies[Title/Abstract])) OR (Cryptogenic Epilepsy[Title/Abstract])) OR (Epilepsies, Cryptogenic[Title/Abstract])) OR (Aura[Title/Abstract])) OR (Auras[Title/Abstract]) | 138,457 |
| 9 | #7 OR #8 | 176,100 |
| 10 | #3 AND #6 AND #9 | 78 |

Embase

No. Query Results

#54 #23 AND #39 AND #53 257

#53 #40 OR #41 OR #42 OR #43 OR #44 OR #45 OR #46 OR #47 OR #48 OR #49 OR #50 OR #51 OR #52 328551

#52 'auras':ab,ti 1336

#51 'aura':ab,ti 12199

#50 'epilepsies, cryptogenic':ab,ti 4

#49 'cryptogenic epilepsy':ab,ti 420

#48 'cryptogenic epilepsies':ab,ti 78

#47 'epilepsy, cryptogenic':ab,ti 17

#46 'epilepsy, awakening':ab,ti 2

#45 'awakening epilepsy':ab,ti 18

#44 'seizure disorders':ab,ti 2861

#43 'seizure disorder':ab,ti 3694

#42 'epilepsies':ab,ti 11971

#41 'epilepsy':ab,ti 179680

#40 'epilepsy'/exp 292082

#39 #24 OR #25 OR #26 OR #27 OR #28 OR #29 OR #30 OR #31 OR #32 OR #33 OR #34 OR #35 OR #36 OR #37 OR #38 190571

#38 'malignant gliomas':ab,ti 7196

#37 'gliomas, malignant':ab,ti 38

#36 'glioma, malignant':ab,ti 99

#35 'malignant glioma':ab,ti 7366

#34 'mixed gliomas':ab,ti 201

#33 'gliomas, mixed':ab,ti 3

#32 'glioma, mixed':ab,ti 0

#31 'mixed glioma':ab,ti 195

#30 'tumors, glial cell':ab,ti 2

#29 'tumor, glial cell':ab,ti 2

#28 'glial cell tumor':ab,ti 13

#27 'glial cell tumors':ab,ti 48

#26 'gliomas':ab,ti 44230

#25 'glioma':ab,ti 76140

#24 'glioma'/exp 173051

#23 #1 OR #2 OR #3 OR #4 OR #5 OR #6 OR #7 OR #8 OR #9 OR #10 OR #11 OR #12 OR #13 OR #14 OR #15 OR #16 OR #17 OR #18 OR #19 OR #20 OR #21 OR #22 1747735

#22 'score, risk factor':ab,ti 15

#21 'risk factor score':ab,ti 288

#20 'risk factor scores':ab,ti 139

#19 'score, risk':ab,ti 1267

#18 'risk score':ab,ti 44574

#17 'risk scores':ab,ti 20613

#16 'populations at risk':ab,ti 3516

#15 'population at risk':ab,ti 5704

#14 'correlates, health':ab,ti 11

#13 'health correlates':ab,ti 589

#12 'social risk factor':ab,ti 95

#11 'risk factors, social':ab,ti 137

#10 'risk factor, social':ab,ti 7

#9 'factors, social risk':ab,ti 9

#8 'factor, social risk':ab,ti 0

#7 'social risk factors':ab,ti 1276

#6 'risk factor':ab,ti 383929

#5 'factor, risk':ab,ti 327

#4 'risk factors':ab,ti 821612

#3 'rrisk factors':ab,ti 3

#2 'risk factors':ab,ti 821612

#1 'risk factor'/exp 1311501

Cochrane library

ID Search Hits

#1 MeSH descriptor: [Glioma] explode all trees 2049

#2 (Glioma):ti,ab,kw OR (Gliomas):ti,ab,kw OR (Glial Cell Tumors):ti,ab,kw OR (Glial Cell Tumor):ti,ab,kw OR (Tumor, Glial Cell):ti,ab,kw 2058

#3 (Tumors, Glial Cell):ti,ab,kw OR (Mixed Glioma):ti,ab,kw OR (Glioma, Mixed):ti,ab,kw OR (Gliomas, Mixed):ti,ab,kw OR (Mixed Gliomas):ti,ab,kw 61

#4 (Malignant Glioma):ti,ab,kw OR (Glioma, Malignant):ti,ab,kw OR (Gliomas, Malignant):ti,ab,kw OR (Malignant Gliomas):ti,ab,kw 659

#5 #1 or #2 or #3 or #4 3147

#6 MeSH descriptor: [Epilepsy] explode all trees 3419

#7 (Epilepsy):ti,ab,kw OR (Epilepsies):ti,ab,kw OR (Seizure Disorder):ti,ab,kw OR (Seizure Disorders):ti,ab,kw OR (Awakening Epilepsy):ti,ab,kw 9442

#8 (Epilepsy, Awakening):ti,ab,kw OR (Epilepsy, Cryptogenic):ti,ab,kw OR (Cryptogenic Epilepsies):ti,ab,kw OR (Cryptogenic Epilepsy):ti,ab,kw OR (Epilepsies, Cryptogenic):ti,ab,kw 57

#9 (Aura):ti,ab,kw OR (Auras):ti,ab,kw 1913

#10 #6 or #7 or #8 or #9 11403

#11 MeSH descriptor: [Risk Factors] explode all trees 33011

#12 (Risk Factors):ti,ab,kw OR (Factor, Risk):ti,ab,kw OR (Risk Factor):ti,ab,kw OR (Social Risk Factors):ti,ab,kw OR (Factor, Social Risk):ti,ab,kw 113718

#13 (Factors, Social Risk):ti,ab,kw OR (Risk Factor, Social):ti,ab,kw OR (Risk Factors, Social):ti,ab,kw OR (Social Risk Factor):ti,ab,kw OR (Health Correlates):ti,ab,kw 7861

#14 (Correlates, Health):ti,ab,kw OR (Population at Risk):ti,ab,kw OR (Populations at Risk):ti,ab,kw OR (Risk Scores):ti,ab,kw OR (Risk Score):ti,ab,kw 90806

#15 (Score, Risk):ti,ab,kw OR (Risk Factor Scores):ti,ab,kw OR (Risk Factor Score):ti,ab,kw OR (Score, Risk Factor):ti,ab,kw 38599

#16 #11 or #12 or #13 or #14 or #15 166973

#17 #5 and #10 and #16 9

Web of science

1 TS=(Glioma) OR TS=(Gliomas) OR TS=(Glial Cell Tumors) OR TS=(Glial Cell Tumor) OR TS=(Tumor, Glial Cell) OR TS=(Tumors, Glial Cell) OR TS=(Mixed Glioma) OR TS=(Glioma, Mixed) OR TS=(Gliomas, Mixed) OR TS=(Mixed Gliomas) OR TS=(Malignant Glioma) OR TS=(Glioma, Malignant) OR TS=(Gliomas, Malignant) OR TS=(Malignant Gliomas) 63,701

2 TS=(Epilepsy) OR TS=(Epilepsies) OR TS=(Seizure Disorder) OR TS=(Seizure Disorders) OR TS=(Awakening Epilepsy) OR TS=(Epilepsy, Awakening) OR TS=(Epilepsy, Cryptogenic) OR TS=(Cryptogenic Epilepsies) OR TS=(Cryptogenic Epilepsy) OR TS=(Epilepsies, Cryptogenic) OR TS=(Aura) OR TS=(Auras) 91,736

3 TS=(Risk Factors) OR TS=(Factor, Risk) OR TS=(Risk Factor) OR TS=(Social Risk Factors) OR TS=(Factor, Social Risk) OR TS=(Factors, Social Risk) OR TS=(Risk Factor, Social) OR TS=(Risk Factors, Social) OR TS=(Social Risk Factor) OR TS=(Health Correlates) OR TS=(Correlates, Health) OR TS=(Population at Risk) OR TS=(Populations at Risk) OR TS=(Risk Scores) OR TS=(Risk Score) OR TS=(Score, Risk) OR TS=(Risk Factor Scores) OR TS=(Risk Factor Score) OR TS=(Score, Risk Factor) 1,238,108

4 #3 AND #2 AND #1 138

WanFang

1 主题（脑胶质瘤+神经上皮性肿瘤+胶质瘤+神经上皮肿瘤+脑神经胶质瘤+神经外胚层肿瘤+神经胶质细胞瘤）30000

2 主题 (癫痫+羊癫疯+羊角风+癫痈+癫痛+癫癎+羊癫风+癫病） 20000

3 主题 (危险因素) 120938

4 1 and 2 and 3 93
